# The influence of APACHE II score on the average noise level in an intensive care unit: an observational study

**DOI:** 10.1186/s12871-015-0019-7

**Published:** 2015-03-31

**Authors:** Munhum Park, Pieter Vos, Björn NS Vlaskamp, Armin Kohlrausch, Annemarie W Oldenbeuving

**Affiliations:** 1 Brain, Cognition & Perception Group, Philips Research, High Tech Campus 36, AE Eindhoven, 5656 The Netherlands; 2 Department of Intensive Care Medicine, St. Elisabeth Hospital, P.O. Box 90151, LC Tilburg, 5000 The Netherlands; 3 Human-Technology Interaction Group, Technische Universiteit Eindhoven, P.O. Box 513, MB Eindhoven, 5600 The Netherlands

## Abstract

**Background:**

Noise levels in hospitals, especially in intensive care units (ICUs) are known to be high, potentially affecting not only the patients’ well-being but also their clinical outcomes. In an observational study, we made a long-term measurement of noise levels in an ICU, and investigated the influence of various factors on the noise level, including the acute physiology and chronic health evaluation II (APACHE II) score.

**Methods:**

The average noise level was continuously measured for three months in all (eight) patient rooms in an ICU, while the patient data were also registered, including the APACHE II score. The 24-hour trend of the noise level was obtained for the patients of length-of-stay (LOS) ≥1 day, which was compared to the timeline of the ICU routine events. For the patients with LOS ≥4 days, the average noise levels in the first four days were analyzed, and regression models were established using the stepwise search method based on the Akaike information criterion.

**Results:**

Features identified in the 24-hour trends (n = 55) agreed well with the daily routine events in the ICU, where regular check-ups raised the 10-minute average noise level by 2~3 dBA from the surrounding values at night, and the staff shift changes consistently increased the noise level by 3~5 dBA. When analyzed in alignment with the patient’s admission (n=22), the daytime acoustic condition improved from Day 1 to 2, but worsened from Day 2 to 4, most likely in relation to the various phases of patient’s recovery. Regression analysis showed that the APACHE II score, room location, gender, day of week and the ICU admission type could explain more than 50 % of the variance in the daily average noise level, *L*_*Aeq*,24*h*_. Where these factors were argued to have causal relations to *L*_*Aeq*,24*h*_, the APACHE II score was found to be most strongly correlated: *L*_*Aeq*,24*h*_ increased by 1.3~1.5 dB when the APACHE II score increased by 10 points.

**Conclusions:**

Patient’s initial health condition is one important factor that influences the acoustic environment in an ICU, which needs to be considered in observational and interventional studies where the noise in healthcare environments is the subject of investigation.

## Background

Due to the around-the-clock patient-care activities and numerous life-supporting devices that generate alarm sounds and operational noises, noise levels in hospitals, especially in intensive care units (ICUs) are known to be high [1-4], potentially affecting not only the patient’s comfort and well-being but also the outcome of patient treatment [5,6]. Furthermore, the adverse acoustic condition in ICUs is considered to be one of the risk factors that contribute to the occurrence of ICU delirium, especially in the early period of ICU admission [7]. For more in-depth summaries of the effects of noise on patients, readers are referred to, for example, Xie et al. [8], Hsu et al. [9] and Konkani and Oakley [10].

Given the potentially negative effects of noise on patients, numerous acoustic surveys have been carried out in ICUs and other areas of hospitals. However, the measurement protocols are not always clearly described in the literature, varying considerably from one study to another [11], where the methods used for data analysis also differ between studies, which do not always comply with general conventions. Furthermore, the measurement periods of the acoustic surveys reported in the literature typically range from a few hours to a few days only [3,12-16] at a time, and extremely short measurements (1~15 min) are not uncommon [17-20].

More importantly, most of the previous acoustic measurements were carried out in the context of estimating the impact of unwanted noise on patients’ well-being and clinical outcomes. On the other hand, it is also a question whether patients’ initial condition can influence the overall noise level, which has hardly been investigated in previous studies: A recent study reported by MP and AK showed that, excluding the ‘patient-involved’ noise, the ICU staff’s speech and other activity noises accounted for more than 50 % of the acoustic energy in a single ICU room [4], which may heavily depend, for example, on the level of patient care, thus the patient’s health condition.

In the current study, acoustic measurements were taken for a relatively long period (~3 months) simultaneously in all patient rooms in a single ICU. Given the extensive set of data, 24-hour trends of the noise levels were obtained and analyzed in comparison to the daily routine events in the ICU. Furthermore, the influence of potentially relevant factors on the average noise level was investigated by establishing multiple linear regression models, where the independent variables included, for example, the time since ICU admission, day of week, room location, patient’s gender and the acute physiology and chronic health evaluation II (APACHE II) score [21].

## Methods

### Average noise level

**Figure 1:**
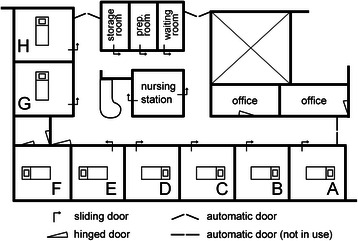
**Layout of ICU.** The layout of the intensive care unit at St. Elisabeth Hospital in Tilburg, The Netherlands, where the current study was carried out. All patient rooms numbered A to H had windows facing outside. Room F was an isolation room with pressure control facility in the ante room. ‘Prep. room’ stands for medication preparation room.

A continuous measurement of the noise levels was carried out in one of the ICUs at St. Elisabeth Hospital in Tilburg, The Netherlands from September to November, 2012. In the ICU, there were 8 single-bed patient rooms along an L-shaped corridor where offices and a nursing station were located on the opposite side of the patient rooms, as shown in Figure 1. One part of the nursing station was an open space with a counter, and most of the sliding doors in the unit (both patient rooms and nursing station) were usually kept open for the observation of the patients. Among the three entrances in the ICU, the one next to Room H was used most frequently, whereas the entrance next to Room A was not in use. Room F was an isolation room with an ante room for pressure control, and when in use for the isolation, the hinged door was obviously kept closed.

In each patient room (dimensions in w ×*d*×h: ~5 ×5×3 *m*^3^), a measurement microphone (M23, Earthworks Inc.) was mounted to the suspended ceiling above the patient bed to the head side by using a pair of nylon cords and a microphone holder. The microphone was positioned 140 cm from the wall behind the patient bed, 60 cm from the suspended ceiling, and 240 cm above the floor. With the patient bed at the default height, the vertical distance between the microphone and the patient’s head was approximately 150 cm in all rooms. The microphone signal was captured at 44.1-kHz sample rate by using a soundcard (M-Audio Fasttrack II, inMusic Brands Inc.) connected to a computer (Nettop nT-535, Foxconn Electronics Inc.). With these hardware components, the upper limit of the measurement was ~125 dB for a test tone at 1 kHz, where the lower limit (noise floor) was ~25 dBA, as measured in an acoustically-treated (anechoic) room^a^. The microphone was calibrated on-site before and after the measurement using a Brüel & Kjaer (B&K) 4231 calibrator.

Using the measurement system described in the preceding text, a custom-made software running on the computer calculated and saved the A-weighted energy-equivalent sound pressure level, *L*_*Aeq*,*T*_, from the captured audio signal, whereas the raw audio signal was discarded immediately. According to the relevant standards [22], *L*_*Aeq*,*T*_ is defined as the total A-weighted acoustic energy divided by the total measurement time, *T*, represented in the unit of decibel (dB) with respect to the reference sound pressure level^b^. The A-weighted energy-equivalent sound pressure level (*average noise level*, hereinafter) is a standard measure widely used to quantify the noise dose in residential areas, offices, schools and healthcare environments, where the measurement period *T* is often prescribed in relevant standards or guidelines, depending on the measurement site and/or the time of day [23]. In the current study, the average noise level was obtained as required for different types of analyses: For the day-by-day analysis, *L*_*Aeq*,24*h*_ was calculated every day since the admission of the patient. Day and nighttime average noise levels were also obtained for 14 hours (7 am - 9 pm) and 10 hours (9 pm - 7 am), respectively. For the 24-hour trend, the average noise level was calculated in a short time frame of 10 minutes, *L*_*Aeq*,10*m*_, from which the arithmetic mean was first obtained within each patient during his/her ICU stay, and then averaged across patient.

### Patient data

For the patients with the length of stay (LOS) equal to or greater than 4 days, the following information was retrieved from the patient data management system (PDMS): Patient’s room number, gender, age, ICU admission type, and admission time. In addition, the APACHE II score was calculated based on the patient data stored in the PDMS. Admission type characterizes whether the patients were admitted to the ICU due to accident (trauma), for medical condition (medical) or after surgery (surgery), whereas the APACHE II score indicates the severity of disease classification [21], ranging from 0 to 71: a higher score corresponds to a more severe condition, thus correlated to higher mortality rate.

### Regression analysis

The correlation between patient data and the average noise level was investigated by establishing multiple linear regression models. Where the daily average noise level *L*_*Aeq*,24*h*_ was taken as the dependent variable, the patient data described in the previous section were considered to be independent variables. From the ICU admission time, the following variables were derived and additionally considered to be independent variables: IsWeekend (true if weekend), ICU day count (counted from ICU admission; 1 to 4), and the 2nd and 3rd power of the ICU day count (since these higher order terms may also be related to the noise level; see Results>First four days).

Given these independent variables, a stepwise search method [24] was used to select the relevant variables that can effectively model the daily average noise level. Assuming that the patient condition may be a good predictor of *L*_*Aeq*,24*h*_, APACHE II was considered to be the ‘seed’ variable for the stepwise search, where the search was executed in both directions (adding and removing variables) based on the Akaike information criterion [25]. The regression analysis presented in the current study were carried out by using R [26] and the following R packages: QuantPsyc [27], leaps [28] and visreg [29].

The current study was approved by the Medical Ethical Committee (Medisch Ethische Toetsingscommissie) at St. Elisabeth Hospital (reference number: 2012.074), which waived the requirement for informed consent.

## Results

### Overview

During the measurement period, the ICU rooms were occupied by 106 patients. The mean and the median of patient’s LOS were 3.2 and 1.0 days, respectively with the interquartile range from 0.7 to 3.4, indicating that a small proportion of the patients stayed in the ICU for relatively long periods. The accumulated duration of the occupied periods (thus the equivalent time span of the available data) was 336.8 days. Since the measurement was continuous (with no interruption at the signal sample rate denoted in Methods), acoustic data were also collected during the unoccupied periods between a patient’s discharge and a new admission. After the discharge of a patient, the room was usually cleaned immediately, and supplies were refilled in preparation of the next patient, in which period the noise level tended to be high. To exclude this short period in the analysis of the unoccupied periods, the patient rooms were considered to be completely empty only 3 hours after the discharge time registered in the hospital database, which resulted in 137.5 unoccupied days available for the data analysis.

The average noise level during the entire measurement period was 53.1 dBA (*L*_*Aeq*,336.8*d*_) and 44.2 dBA (*L*_*Aeq*,137.5*d*_) in the occupied and the unoccupied periods, where the day and nighttime average noise levels during the occupied period were 54.2 dBA and 51.1 dBA, respectively. In a good agreement with the usual range of the *L*_*Aeq*_ values (50~60 dBA) as reported by many authors including Darbyshire and Young [1], the average noise level of this particular ICU was also found to be very high, far above the level recommended, for example, by World Health Organization [23] (<35 dB *L*_*Aeq*_). Arguably, there is an urgent need to update relevant guidelines so that many concerning parties (e.g., hospitals, government agencies, and so on) may work together towards a realistic thus achievable target to improve the acoustic conditions in healthcare environments.

### 24-hour trend

**Figure 2:**
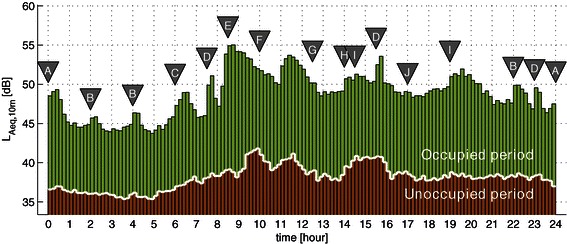
**24-hour trend of the average noise level every 10 minutes (*L***_***Aeq*,10*m***_**).** The trends were averaged over 55 patients (299 occupied days) and over 134 unoccupied days, respectively. Inverted triangles indicate the time stamps of the daily routine events in the ICU, as listed in Table 1.

During the measurement period, 55 patients stayed in the ICU for one day or longer, and 24-hour trends were obtained given the 299-day dataset associated with these patients. As shown in Figure 2, *L*_*Aeq*,10*m*_ varied throughout the occupied days from 43.8 dB to 55.0 dB on average. Where the daily routine in this particular ICU is summarized in Table 1, the peaks and troughs of the average seem to correspond well to the listed events. For example, regular check-ups took place in the ICU every two hours, which show up in the graph prominently at 2, 4 and 22 o’clock, where the check-ups at 0 and 6 o’clock combined with additional patient-care activities (e.g., doctors’ round or X-ray) raised the noise level noticeably higher than the normal check-ups. When the morning routine began, the average noise level increased by ~10 dBA, quickly reaching the daily maximum at 55 dBA, and then gradually decreased in the next few hours. When the staff coffee break ended at 11am, the noise level returned close to the maximum, again gradually decreasing towards the lunch break. On average, regular check-ups during the nighttime raised *L*_*Aeq*,10*m*_ by 2~3 dB from the surrounding values, and similarly the staff shift changes at 7.30, 15.30 and 23 o’clock consistently increased the 10-minute average noise level by 3~5 dB for approximately 20 minutes.

**Table 1:** **An overview of the daily routine events in the ICU at St. Elisabeth Hospital**

Label	Time (hour)	Events
A	0	Check-up with doctors’ round
B	2; 4; 22	Check-up (every 2 hours)
C	6	Check-up with X-ray
D	7.30; 15.30; 23	Nursing shift change
E	8.30	Morning routine begins
F	10	Staff coffee break (ends at 11)
G	12.30	Staff lunch break begins
H	14	Check-up (patients turned)
I	14.30; 19	Visiting hour begins
J	17	Dinner begins

Morning routine (E) includes: Doctors’ round, washing patients, physiotherapy, etc.

When the room was empty, *L*_*Aeq*,10*m*_ varied considerably less than during the occupied period [see Figure 2]. There were a few noticeable peaks and troughs in the graph, which however were difficult to relate to the specific events in the ICU.

In the literature, similar attempts have been made to show the 24-hour trend of the noise level in ICUs (see, e.g., Aitken [16]). Due to the limited periods of acoustic measurements (typically a few days) thus high variability, however, it was difficult to relate the features in the noise-level trend (e.g. peaks or troughs) to the ICU workflow. As a matter of fact, the 24-hour trends obtained from a relatively large set of data in the current study may provide valuable information about the average noise levels of particular ICU events and activities, which could help hospital staff to take appropriate actions to reduce the noise level in the ICU. On the other hand, it should be recalled that these trends were obtained by averaging the time-aligned data over many days, and therefore the contribution of temporally inconsistent sound events (e.g., alarm sounds) were averaged out, which however may also significantly influence the overall noise level (see Park et al. [4]).

### First four days

The number of patients with LOS ≥4 was 22, for whom the associated patient data were retrieved or derived from the PDMS, as summarized in Table 2. Accordingly, the noise-level data corresponding only to these patients for the first four days (equivalent to 88 days = 22 patients × 4 days) were further analyzed in the following sections.

**Table 2:** **List of the independent variables considered for the regression analysis**

Independent variables		Levels
Per patient	Room number	A(1), B(3), C(4), D(2), E(2), F(2), G(6) and H(2)
	Gender	Male(9) and female(13)
	Age	34~80; mean 57.3
	ICU admission type	Medical(10), surgery(9) and trauma(3)
	APACHE II	9~34; mean 19.0
Per day	IsWeekend	Weekday(69) and weekend(19)
	Day count	1,2,3 and 4
	(Day count) ^2^	1,4,9 and 16
	(Day count) ^3^	1,8,27 and 64

The number in parenthesis for each level indicates the number of the corresponding patients and samples for the per-patient and per-day variables, respectively.

**Figure 3:**
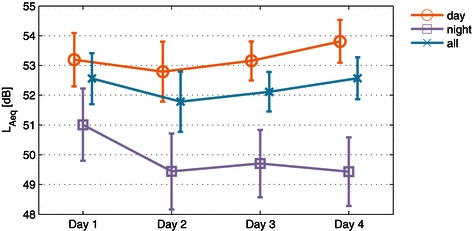
**Average noise levels in the first four days.** Means and 95-% confidence intervals are shown for *L*_*Aeq*_ averaged over 22 patients. Daytime was defined to be between 7 am and 9 pm (therefore, *L*_*Aeq*,14*h*_ for daytime, *L*_*Aeq*,10*h*_ for nighttime).

Figure 3 shows the trends of the average noise level since the patients’ admission to the ICU. For the daytime average, the noise level decreased from Day 1 to Day 2 by ~0.5 dB, and then increased to Day 3 and 4, although the range of the overall change was only within ~1 dB. On the other hand, the nighttime average generally decreased from Day 1 to Day 4 in the range of ~1.5 dB. A two-way repeated-measure ANOVA indicated that the effect of day was significant [ *F*(3,63)=3.404; *p*=.023], and so was that of the time of day [day/night; *F*(1,21)=96.5; *p*<.001]. A post-hoc analysis was carried out by comparing Day 1 to Day 2 and Day 2 to Day 4 based on paired t-tests, which showed that the average noise level on Day 1 differed from Day 2 during the nighttime (*p*=.008), and Day 2 from Day 4 during the daytime (*p*=.005), which were significant with an appropriate Bonferroni correction (*p*<.025).

The data presented in Figure 3 suggest that the daytime acoustic condition in the ICU may generally improve from Day 1 to Day 2, but worsen in the following days, whereas the nighttime noise level may continue decreasing. Without the detailed information of the sources of noise, it is difficult to understand the actual causes of this trend, which however may be interpreted in relation to different stages of patient care and recovery: On admission to the ICU, initial check-ups and urgent treatments are carried out by the medical staff, which tend to raise the noise level in the first 24 hours regardless of day or nighttime. As the patient condition stabilizes, patient-care activities may decrease, thus lowering the noise level. However, patients whose conditions are improving may begin interacting with the ICU staff or visitors, especially during the day, which may increase the daytime noise level again. Nevertheless, it is difficult to argue how the daily trend may affect patients’ comfort and the quality of their rest or sleep, unless the patient’s own contribution is clearly separated.

The characteristics of the noise levels on the first days of the ICU admission may be subject to further research, where the environmental condition in the early period of patient’s ICU stay is often hypothesized to correlate to the occurrence of ICU delirium [7].

### Regression analysis

As summarized in Table 3, five independent variables were found by the stepwise search method to explain 53.1 % of the variance in the daily average noise level: APACHE II, room number, admission type, gender, and IsWeekend, as denoted in the step-3 model listed in Table 3, where two additional hierarchical models are also summarized. Among the single-variable regression models, the model with APACHE II explained the variance of *L*_*Aeq*,24*h*_ to the largest extent (*R*^2^=0.216; step 1), which agrees well with the initial assumption made for the stepwise search. When the room number was added, the two-variable model could explain 44.1 % of the variance (step 2).

**Table 3:** **Three hierarchical regression models for the daily average noise level (** ***L***_***Aeq,24h***_ **)**

		*R* ^2^	Adjusted*R*^2^	B [SE B] (beta)	*p*
Step 1		0.216	0.207		
	Constant			49.729 [0.548]	<.001^∗^
	APACHE II			0.133 [0.027] (0.465)	<.001^∗^
Step 2		0.441	0.384		
	Constant			51.152 [0.716]	<.001^∗^
	APACHE II			0.146 [0.026] (0.511)	<.001^∗^
	Room number			- [-] (-)	<.001^∗^
Step 3		0.531	0.457		
	Constant			51.768 [1.039]	<.001^∗^
	APACHE II			0.140 [0.026] (0.490)	<.001^∗^
	Room number			- [-] (-)	<.001^∗^
	IsFemale			1.444 [0.566] (5.042)	.013^∗^
	IsWeekend			-0.688 [0.374] (-0.816)	.070
	ICU admission type			- [-] (-)	.103

The step-3 model was first established by selecting independent variables based on the Akaike information criteria, whereas the subsets of the selected five variables were used for the other two models. (B: Coefficient value; SE B: Standard error of the coefficient value; beta: Normalized coefficient value).

**Figure 4:**
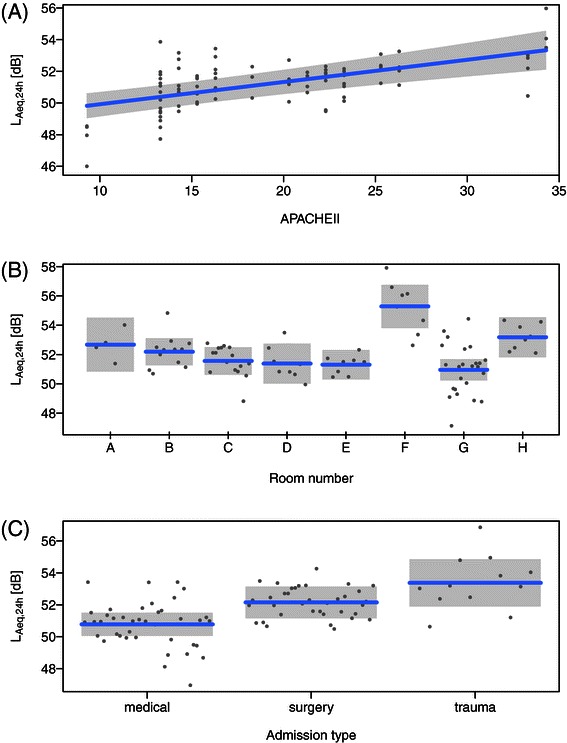
**Results of the regression analysis.** Regression lines are shown for **A)** APACHE II, **B)** room number and **C)** admission type for the individual data samples indicated as gray dots. Shaded areas indicate the 95- % confidence interval (band). All figures were produced by using visreg [29].

Given the regression coefficients listed in Table 3, *L*_*Aeq*,24*h*_ increased by 1.3~1.5 dB when the APACHE II score increased by 10 points. As shown in Figure 4(A), the APACHE II scores of the patients observed in the current study ranged from 9 to 34 points, for which the daily average noise level varied approximately from 50 to 53 dBA. Although it may be seen as a small difference, the 3-dBA increase in logarithmic scale is equivalent to a doubling of the acoustic energy (see the definition of *L*_*Aeq*,*T*_ in Endnotes), which may result from the doubling of the number (or the duration) of noisy events, thus potentially affecting the patient’s sleep/rest quality.

Room number is a multilevel variable, for which the coefficients were given to the associated dummy variables used in the regression analysis, and therefore its influence on the daily average noise level is better illustrated in Figure 4(B). In general, the rooms closer to the nursing station were slightly quieter, which suggests that the sources of noise at the nursing station (e.g. speech activities by staff) may not be so influential to the acoustic conditions in patient rooms. Where the frequent operation of the automatic door at the main entrance may have resulted in a relatively high noise level in Room H, the remarkably high values of *L*_*Aeq*,24*h*_ in the isolation room (Room F) [see Figure 4(B)] suggests that the acoustic condition in the patient room was influenced predominantly by the noise sources within the room which are closely linked to the condition of patient.

Although the stepwise search resulted in the selection of five independent variables, including admission type, gender, and IsWeekend, the addition of these three variables contributed to the increase of the *R*^2^ value only by ~0.09 from step 2 to 3, where the corresponding p-values were higher (thus less significant) than those for the two other variables, APACHE II and room number (see Table 3). Nevertheless, it is interesting to note that the regression coefficient for gender was significant, where the noise level was higher for female patients by ~1.4 dBA. Although not significant, the trends of the noise level corresponding to the remaining two variables, IsWeekend and admission type seem to agree with general expectations: Weekends are quieter than weekdays; the noise levels for the patients admitted for ‘unexpected’ accidents are generally higher than those for the patients after ‘planned’ surgeries or for medical conditions [see Figure 4(C)].

Finally, it is noteworthy that, despite the clear trend observed in Figure 3, the day count since ICU admission and its higher order terms were not selected as the predictors of the daily average noise level based on the Akaike information criterion, neither was the age of the patient.

## Discussion

The results of the regression analysis summarized in Table 3 only suggest that the noise level is ‘related’ to many variables specific to the patient (APACHE II, admission type and gender), time (weekend/weekday) and the location of the room (room number). Excluding the APACHE II score, however, it is clear that the remaining four variables have ‘causal relations’ to the noise level, as these variables are fixed, and may not be influenced by the acoustic condition. Furthermore, the relation of APACHE II to the noise level may also be causal given the following arguments:

- APACHE II is a predictive score given within the first 24 hours of ICU admission, where the laboratory tests required for the calculation of the score (e.g. blood tests) are initiated on patient’s admission [21]. Therefore, it is unlikely that the high noise levels influence the APACHE II score.
- In Figure 4(A), an approximately 3-dBA increase in noise level is shown to relate to a 25-point increase of APACHE II, which is equivalent to the increase of mortality rate by 60~70% [21]. It is unlikely that there exists such an extreme effect of acoustic condition on mortality rate.

Therefore, the results of the regression analysis presented in the previous section suggest that many factors, particularly the condition of patients may have causal effects on the acoustic condition, which has the following implications:

1. For observational studies where the effects of noise on patient’s condition are investigated, it is essential to understand that the patient’s initial and/or existing conditions may influence the characteristics of the acoustic environment. Therefore, great care has to be taken to isolate the additional and exclusive effects of noise on patient’s clinical outcome.
2. In the literature, a number of studies report the results of interventions to reduce the noise levels in hospitals, where the examples of the intervention include the acoustic treatment of patient/staff areas [30] and the staff education occasionally accompanied by the use of noise feedback/monitoring devices [31-33]. Typically in these studies, acoustic measurements are carried out two times before and after an interventional scheme, of which the results are compared to evaluate the effectiveness of the scheme. Given the fact that the patient condition and various other factors may influence the acoustic environment, these factors have to be carefully controlled or balanced in the two measurement periods, which is unfortunately not the case in all studies in the past (see, e.g., Chang et al. [33]).

### Limitations of the study

The regression models presented in the current study were established based on the data obtained for the patients with LOS ≥4 days, whose characteristics may differ from those of general ICU patients. Also, the ranges of some independent variables were limited in the current study. Therefore, the results of the regression analysis may not readily be generalized. For example, the relation between the daily average noise level and the APACHE II score may not be extrapolated beyond 34 points.

## Conclusions

In the current study, acoustic measurements were carried out in 8 patient rooms in an intensive care unit for 3 months. Given a relatively large set of data, the effects of various factors were analyzed on the average noise level. For example, the time of the day influenced the average noise level, where the 24-hour trend was found to correspond well to the daily routine events in the ICU (e.g., regular check-ups, shift changes, visiting hours, and so on). It was also shown that the acoustic condition may vary in the first few days, arguably according to the phases of patient treatment and recovery.

In the regression analysis, the daily average noise level was found to vary with the severity of disease (APACHE II), room location, gender, day of week, and ICU admission type, where the highest correlation was found with the APACHE II score. It was also argued that these independent variables have causal relations to the average noise level.

The findings of the current study suggest that the patient characteristics among others can be important factors that influence the acoustic environment in ICUs, and therefore must be carefully considered in observational and interventional studies where the noise in healthcare environments is the subject of research.

## Key messages

- The 24-hour trend of average noise level corresponded well to the routine events in the ICU.
- The average noise level in ICU varied in the first few days since the patient’s admission, in a likely relation to the different phases of patient treatment and recovery.
- Many factors can be modifiers of the average noise level in ICU, among which the APACHE II score was shown to be most dominant. These factors must be taken into account in studies designed to quantify the effects of noise or noise reduction scheme.

## Endnotes

^a^ Noise level is represented in decibel (dB) with respect to the reference sound pressure, *p*_0_=20 *μ*Pa. Sound pressure is often ‘A-weighted’ in spectrum before being compared to *p*_0_ [22], roughly reflecting the frequency dependency of human hearing sensitivity, which is indicated by a suffix to the unit: dBA or dB(A).

^b^ The A-weighted energy-equivalent sound pressure level in a given time period, *T* can be represented as follows [22]: $ L_{Aeq,T}=10\log _{10} \left (\frac {1}{T}{\int ^{T}_{0}} \left (\frac {p_{A}(t)}{p_{0}} \right)^{2} dt \right), $ where *p*_*A*_(*t*) is the A-weighted sound pressure as a function of time, and *p*_0_ is the reference sound pressure, *p*_0_=20 *μ*Pa. The square of *p*_*A*_(*t*) integrated over a certain time is proportional to the total acoustic energy.

## Abbreviations

APACHE II: Acute physiology and chronic health evaluation II
ICU: intensive care unit
LOS: Length of stay
PDMS: Patient data management system
